# Psychological states and needs among post‐allogeneic hematopoietic stem cell transplantation survivors

**DOI:** 10.1002/cam4.6280

**Published:** 2023-06-27

**Authors:** Sun Yanling, Yan Pu, Xu Duorong, Zhong Zhiyong, Xu Ting, Xin Wenjun, Zhang Xiangzhong, Zhang Jingwen

**Affiliations:** ^1^ Department of Hematology The Third Affiliated Hospital of Sun Yat‐sen University Guangzhou China; ^2^ Department of Hematology The First Affiliated Hospital of Sun Yat‐sen University Guangzhou China; ^3^ Department of Psychiatry The Third Affiliated Hospital of Sun Yat‐sen University Guangzhou China; ^4^ Zhongshan School of Medicine Sun Yat‐sen University Guangzhou China; ^5^ Zhongshan School of Medicine and Guangdong Province Key Laboratory of Brain Function and Disease Sun Yat‐sen University Guangzhou China

**Keywords:** allogeneic hematopoietic stem cell transplantation, comorbidities, demands and needs, depression, sleep quality

## Abstract

**Objectives:**

To analyze psychological states and needs of patients after allogeneic hematopoietic stem cell transplantation (allo‐HSCT).

**Methods:**

Questionnaires were sent to 101 allo‐HSCT survivors and 96 questionnaires were returned. The questionnaire covered several categories: (1) demographics and general information, (2) physical conditions, (3) psychologic status and sleep quality, (4) survivor's comments on transplantation, (5) demands and needs, (6) preferred forms and channels of information.

**Results:**

Depression and poor sleep quality were major concerns troubling allo‐HSCT survivors. A notable discrepancy exists between clinically diagnosed depression (4.2%) and self‐ reported depression based on BDI‐13 (55.2%). Young adults (18–49 years old), chronic graft‐versus‐host disease, the ECOG performance score of 2–4, surviving within 5 years since HSCT, no or low dose of ATG used and being single were significantly associated with self‐reported depression. Based on the PSQI score, 75% of survivors experienced varying degrees of sleep quality impairment. Young adults, chronic GVHD and the ECOG score of 2–4 were significantly associated with worse sleep quality. The majority of patients reported unmet needs on physical and psychosocial aspects. Nutrition information was the most important topic, followed by disease treatments and fatigue. Differences in informational needs were found in the survivors according to age, time since HSCT and gender. WeChat public account and WeChat applet, mobile interactive platform and one‐to‐one conversation were the favorite channel for information.

**Conclusions:**

Clinicians should establish more appropriate survivorship care plans focusing on survivors' psychologic states, demands and needs.

## 1 BACKGROUND

Allogeneic hematopoietic stem cell transplantation (allo‐HSCT) is commonly used as a curative therapy for hematological malignancies. Along with advances in prevention and treatment of complications, the number of survivors post‐HSCT is steadily increasing. However, the recovery process after allo‐HSCT is long‐term and often accompanied by psychological and physical issues.^1^, ^2^

This study was prompted by a case of suicide by a patient in our center who had undergone allo‐HSCT. Further investigation^3^ revealed higher suicide rates among HSCT patients compared to the general population. Additionally, many allo‐HSCT survivors require psychotherapy,^4^, ^5^ and even those who do not meet diagnostic criteria may still experience distress.

A few studies on the associations among predictors, physical and mental recovery from transplant, long‐term psychosocial functioning, and quality of life have yielded conflicting results.^6^, ^7^, ^8^, ^9^, ^10^ It should be noted that most studies examined patients including both auto‐HSCT and allo‐HSCT.^1^, ^5^, ^11^ However, preconditioning regimen, post‐HSCT complications, and immune reconstitution are much different between them. Compared with auto‐HSCT, allo‐HSCT survivors have much more significant risks and burdens due to more severe complications such as GVHD. Therefore, psychological impairments of allo‐HSCT survivors are considered more serious. So far, there has been limited experience on psychological states and needs of Allo‐HSCT survivors, potentially leading to delayed identification and intervention for those experiencing psychological deficits.

In this study, we present the results of an analysis performed in allo‐HSCT survivors, focusing on psychologic states, concerns and needs, taking into account transplant‐specific factors.

## 2 MATERIALS AND METHODS

### 2.1 Study design and patients

The survivors were recruited from the Transplantation Center of the Third Affiliated Hospital of Sun Yat‐sen University, who had undergone allo‐HSCT between 2012 and 2022. Patients who did not have survey data, had a relapse, failed to be contacted, or underwent autologous HSCT were excluded. According to this, 101 questionnaires were sent to the survivors. Clinical data were obtained from the case record. The study was approved by the local ethics committee. Participants were recruited through telephone and outpatient visits. Potential participants were contacted by Wechat with a letter inviting them to participate in this study exploring the psychosocial issues they might have faced following allo‐HSCT. Those who were interested in participating would provide written informed consents and fill in WeChat‐based electronic questionnaires online. The study was registered at the Chinese Clinical Trial Registry (www.chictr.org) (Identifier: ChiCTR 2200057497).

### 2.2 Questionnaire

The questionnaire includes Beck Depression Inventory 13 (BDI‐13), Pittsburgh sleep quality index (PSQI), ECOG and some custom questions.

Beck Depression Inventory (BDI) is one of the most widely used self‐report measures to assess depressive symptom severity. The original version was developed in 1961 and underwent two major revisions in 1978 and 1996, as the BDI‐IA and BDI‐II respectively.^12^, ^13^ All the three versions consist of 21 items. A. T. Beck and R. W. Beck developed the short form of the BDI, the BDI‐13. Correlations of 0.94 or higher was found between BDI‐13 and BDI‐21,^14^ suggesting the BDI ‐13 is an acceptable substitute for BDI ‐21. BDI‐13 is a self‐report measure comprised of 13 items reflecting specific cognitive, affective, and physical symptoms of depression, using four alternatives varying from “rarely” through “often” and corresponding statements scored from 0 to 3. The sum of all the item scores yields the BDI total score. The following norms were proposed: normal (0–4); mild depression (5–7); moderate depression (8–15); and severe depression (≥16).

The sleep quality is measured by PSQI.^15^ PSQI Scale consists of 9 questions and derived from 7 assessment components such as sleep latency, sleep duration, sleep efficiency, sleep disturbance, use of medication, and daytime dysfunction, representing the past month. PSQI score ranging from 0 to 21, with higher scores indicating worse sleep quality.

To optimize our information offer, we created some custom questions examining psychological status and evaluating the needs of HSCT survivors. The questions consisted of the following principal categories: (1) demographics and general information, (2) physical conditions, (3) psychologic status, (4) survivor's comments on transplantation, (5) demands and needs, and (6) preferred forms and channels of information. Among them, the questions associated with “demands and needs” consisted of 16 items in four major categories: (1) do you think the recipients need more help, including financial, psychological and living aspects? (2) What kind of support do you expect from the doctor? (3) What are your medical and psychosocial informational needs? (4) What's your preferred channel of information. The 13 Items about need for advice on medical and psychosocial aspects are rated on a five‐point Likert scale, ranging from 1 (very low) to 5 (most intensive need).

The questionnaires were filled out respectively on line and then returned researchers automatically.

### 2.3 Statistical analyses

With an prevalence rate of mental diseases such as depression, anxiety, sleep disorders between 5%–19% among allo‐HSCT survivors, the sample size should be *n* = 73 to 237 by calculation. Therefore 101 is within a reasonable range. Data analysis was performed on all returned questionnaires. Continuous data are expressed as means ± standard deviation and skewed data are presented as medians and interquartile range. All categorical data were presented as a percentage or an absolute number. Group comparisons were performed using the Chi‐square test and the Mann–Whitney *U*‐test. Calculations were performed using IBM SPSS Statistics Version 23. For all analyses, two‐sided *p* < 0.05 was considered statistically significant.

## 3 RESULTS

### 3.1 Participant information

96 of the 101 (95%) questionnaires were returned and eligible for evaluation. Table 1 presents the patient and clinical characteristics; the median age was 35 years (range, 13–60). Acute myeloid leukemia, acute lymphoblastic leukemia were noted in 51%, 30.2% patients, respectively. Standard dose of anti‐thymocyte globulin was used in 71.9% of patients. The time since allo‐HSCT was 4.2 years (range 0.3–12). These recipients had a range of complications and comorbidities. Chronic graft‐versus‐host disease (cGVHD) was the most common (33.3%, *n* = 32). More than 10% individuals suffered from the following diseases: pulmonary disease, frequent infection, osteoarthritis, gastrointestinal diseases, back pain, and anemia.

**TABLE 1:** Baseline characteristics of patients and the comorbidities (*n* = 96).

Clinical factors	No. (%)
Number (male/female)	96 (47/49)
Age, median (range), years	35 (13–59)
Marital status	
Single	44 (45.8)
Married	46 (47.9)
Divorced/widowed	6 (6.3)
Education	
High school or below	53 (55.2)
Junior college or university	41 (42.7)
Graduate school	2 (2.1)
Diagnosis	
AML	49 (51.0)
ALL	29 (30.2)
MDS	6 (6.3)
PNH/AA	6 (6.3)
CML	4 (4.2)
CMML	1 (1.0)
PMF	1 (1.0)
Donor	
Matched sibling	32 (33.3)
Unrelated	19 (19.8)
Haploidentical family	42 (43.8)
Unrelated cord blood	3 (3.1)
Conditioning regimen	
BuCy	87 (90.6)
With TBI	4 (4.2)
Others	5 (5.2)
ATG	
Standard dose	69 (71.9)
Low dose	9 (9.4)
No	18 (18.8)
Second transplantation	4 (4.2)
Time since HSCT, years	
0–0.5	6 (6.3)
0.5–1	9 (9.4)
1–2	23 (24.0)
2–5	21 (21.9)
>5	35 (38.5)
Complications and comorbidities	
Chronic GVHD	32 (33.3)
Hepatitis B	9 (9.4)
Tuberculosis	3 (3.1)
Frequent infection	10 (10.4)
Heart disease	3 (3.1)
Hypertension	5 (5.2)
Pulmonary disease	21 (21.9)
Diabetes	3 (3.1)
Gastrointestinal diseases	15 (15.6)
Renal disease	6 (6.3)
Liver disease	7 (7.3)
Anemia	10 (10.4)
Other malignancy disease	2 (2.1)
Depression (self‐consciousness)	4 (4.2)
Osteoarthritis	16 (16.7)
Back pain	14 (14.6)
Eye disease	4 (4.2)

Abbreviations: ALL, acute lymphoblastic leukemia; AML, acute myeloid leukemia; ATG, anti‐thymocyte globulin; BuCy, busulfan plus cyclophosphamide; CML, chronic myeloid leukemia; CMML, chronic myelomonocytic leukemia; GVHD, graft‐versus‐host disease; MDS, myelodysplastic syndrome; PMF, primary myelofibrosis; PNH/AA, paroxysmal‐nocturnal‐hemoglobinuria/aplastic anemia; TBI, total body irradiation.

### 3.2 Psychologic status, sleep quality, and general health state

Table 2 presents the psychologic status, sleep quality and general health state.

**TABLE 2:** Evaluation of psychologic status, sleep quality, and general health state: self‐evaluated depression, BDI‐13, Pittsburgh sleep quality index (PSQI), self‐evaluated health state BMI, and ECOG in hematopoietic stem cell transplantation survivors.*, p<0.05; **, p<0.005

	Total (%)	Male (%)	Female (%)	*p* value
Patients	96	47	49	
Clinically diagnosed depression	4 (4.2)	2 (4.3)	2 (4.1)	1.000
Self‐reported depression (BDI)				
Healthy (0–4)	43 (44.8)	23 (49.0)	20 (40.8)	0.424
Mild (5–7)	28 (29.2)	10 (21.3)	18 (36.7)	0.096
Moderate (8–15)	20 (20.8)	12 (25.5)	8 (16.3)	0.267
Severe (≥16)	5 (5.2)	2 (4.3)	3 (6.1)	0.681
Sleep quality (PSQI)				
Global PSQI, 0–3	24 (25.0)	15 (31.9)	9 (18.4)	0.125
Global PSQI, 4–8	41 (42.7)	18 (38.3)	23 (46.9)	0.392
Global PSQI, 9–16	28 (29.2)	13 (27.7)	15 (30.6)	0.750
Global PSQI, ≥17	3 (3.1)	1 (2.1)	2 (4.1)	0.582
BMI, kg/m^2^				
Low body weight (BMI <18.5 kg/m^2^)	30 (31.3)	8 (17.0)	22 (44.9)	0.003**
Normal weight (BMI 18.5–23.9 kg/m^2^)	53 (55.2)	32 (68.1)	21 (42.9)	0.013*
Overweight (BMI 24–27.9 kg/m^2^)	10 (10.4)	6 (12.8)	4 (8.2)	0.520
Obesity (BMI ≥28 kg/m^2^)	3 (3.1)	1 (2.1)	2 (4.1)	1.000
ECOG				
ECOG = 0	25 (26.0)	14 (29.8)	11 (22.5)	0.413
ECOG = 1	59 (61.5)	27 (57.5)	32 (65.3)	0.429
ECOG = 2	9 (9.4)	3 (6.4)	6 (12.2)	0.487
ECOG = 3	2 (2.1)	2 (4.3)	0 (0.0)	0.237
Health state (self‐evaluation)				
Very good	9 (9.4)	8 (17.0)	1 (2.0)	0.012*
Good	53 (55.2)	28 (59.6)	25 (51.0)	0.399
Unsatisfied	30 (31.3)	9 (19.2)	21 (42.9)	0.012*
Poor	4 (4.2)	2 (4.3)	2 (4.1)	1.000
Selfcare ability (self‐evaluation)				
Capable of selfcare	61 (63.5)	34 (72.3)	27 (55.1)	0.079
Sometimes need a caregiver	25 (26.0)	7 (14.9)	18 (36.7)	0.015*
Most time need a caregiver	7 (7.3)	4 (8.5)	3 (6.1)	0.954
Full assisted livings	3 (3.1)	2 (4.3)	1 (2.0)	0.971

There was a significant disparity between clinically diagnosed depression and self‐reported depression based on BDI‐13. Only 4.2% survivors were diagnosed with depression by psychiatrists. However, based on BDI‐13, depression was a major concern troubling them. Mild, moderate, and severe depression accounted for 29.2%, 20.8% and 5.2%, respectively. Female survivors appeared to be more prone to mild depression than male survivors (36.7% vs. 21.3%, *p* = 0.096). 75% survivors reported different degrees of sleep disorders, 33.3% had poor or awful sleep quality.

To objective assess the survivors' health states, we calculated their BMI and inquired their ECOG status. 31.3% survivors were underweight (BMI: <18.5 kg/m^2^). Female survivors seemed to have poorer nutritional status (44.9% vs. 17.0%, *p* = 0.003). Low body weight post‐allo‐HSCT was a noteworthy phenomenon, especially in female survivors. Up to 42.7% survivors complained weights lost (data not shown here). 74% survivors reported varying degrees of physical activity limitations. 9.4% survivors' ECOG score were 2% and 2.1% survivors were 3.

We asked the survivors about their self‐perceived health states and selfcare abilities. In subjective aspects, male survivors held more optimistic attitudes on their health states compared with female (17.0% vs. 2.0%, *p* = 0.012; 42.9% vs. 19.2%, *p* = 0.012). When asked about selfcare ability, 63.5% survivors reported they were capable of selfcare. Lower proportions of male survivors needed living assistants than female survivors (14.9% vs. 36.7%, *p* = 0.015). 7 (7.3%) survivors reported most of the time they needed to be taken care of, and 3 (3.1%) survivors reported they needed a specialized full‐time caregiver.

### 3.3 Differences in depression and sleep quality according to clinical factors

BDI‐13 and PSQI were analyzed respectively in subgroups for clinical factors (Table 3). Young adults (18–49 years old), developing cGVHD, the ECOG score of 2–4 and single survivors had higher BDI‐13 score (*p* = 0.041; *p* = 0.001; *p* = 0.000; *p* = 0.049). Surviving beyond 5 years since HSCT, using standard dose of ATG were associated with lower BDI‐13 score (*p* = 0.035; *p* = 0.033). No significant difference in BDI‐13 score was noted by gender, primary diseases, donor type, conditioning regimen or education levels.

**TABLE 3:** Differences in depression, and sleep quality according to criteria groups (*n* = 96).

	Depression (BDI‐13)		Sleep quality (global PSQI)	
	Median (range)	*p* value	Median (range)	*p* value
Sex		*p* = 0.624		*p* = 0.137
M	5 (1–10)		5 (3–9)	
F	5 (2–7)		7 (4–10)	
Age, year		*p* = 0.041*		*p* = 0.035*
<18	4 (1–7)		2 (2–2)*	
18–49	6 (2–9)*		6 (4–10)*	
≥50	1 (0–3)*		4.5 (2–11)	
Diagnosis		*p* = 0.538		*p* = 0.186
AML, ALL, MDS, CML, CMML	5 (1–8)		6 (3–10)	
PNH, AA, PMF	6 (3.5–8.5)		9 (7–10)	
Donor		*p* = 0.108		*p* = 0.482
Matched sibling	6.5 (3–10)		7 (4.5–10)	
Unrelated donors	4 (1–8)		6 (3–12)	
Haploidentical family	4 (0–7)		5 (2–10)	
Unrelated cord blood	7 (6–8)		5 (5–7)	
Years since HSCT		*p* = 0.035*		*p* = 0.353
0–5	6 (2–9)*		6 (3–11.5)	
≥5	4 (1–7)*		5 (4–8)	
Conditioning regimen		*p* = 0.935		*p* = 0.253
BuCy	5 (1–8)		6 (4–10)	
TBI	6 (3–8)		3.5 (2–7)	
Others	6 (3–6)		10 (8–10)	
ATG		*p* = 0.033*		*p* = 0.416
Standard dose (10 mg/kg)	4 (1–7)*		6 (3–10)	
No or low dose (0 or 3 mg/kg)	7 (3–12)*		6 (5–9)	
Chronic GVHD		*p* = 0.001**		*p* = 0.007*
Yes	7 (4–14.5)*		8.5 (5–12.5)*	
No	4 (1–7)*		5 (3–8)*	
ECOG		*p* = 0.000**		*p* = 0.000**
0–1	4.5 (1–7) *		5 (3–9) *	
2–4	12 (7.5–15) *		13.5 (8.5–15.5) *	
Marital status		*p* = 0.049*		*p* = 0.627
Single/divorced/widowed	6 (3–9) *		6 (4–10)	
Married	3 (1–7) *		6 (3–10)	
Education		*p* = 0.540		*p* = 0.197
High school or below	5 (1–7)		5 (3–9)	
Junior college or university	6 (1–9)		7 (4–11)	
Graduate school	6 (5–7)		3.5 (2–5)	

Abbreviations: AA, aplastic anemia; ALL, acute lymphoblastic leukemia; AML, acute myeloid leukemia; ATG, anti‐thymocyte globulin; BuCy, busulfan plus cyclophosphamide; CML, chronic myeloid leukemia; CMML, chronic myelomonocytic leukemia; GVHD, graft‐versus‐host disease; MDS, myelodysplastic syndrome; PMF, primary myelofibrosis; PNH, paroxysmal‐nocturnal‐hemoglobinuria; TBI, total body irradiation.

*p < 0.05; **p < 0.005.

Higher PSQI scores indicated worse sleep quality. Adolescences (<18 years old) showed lower median PSQI score than young adults (18–49 years old) (2 vs. 6, *p* = 0.035). Developing cGVHD and the ECOG performance score of 2–4 were significantly associated with higher PSQI score (*p* = 0.007; *p* = 0.000). No significant difference in PSQI score was noted by gender, primary disease, surviving time since HSCT, conditioning regimen, ATG using, marital status and education level.

### 3.4 Transplantation evaluation and emotion states post‐allo‐HSCT

**FIGURE 1:**
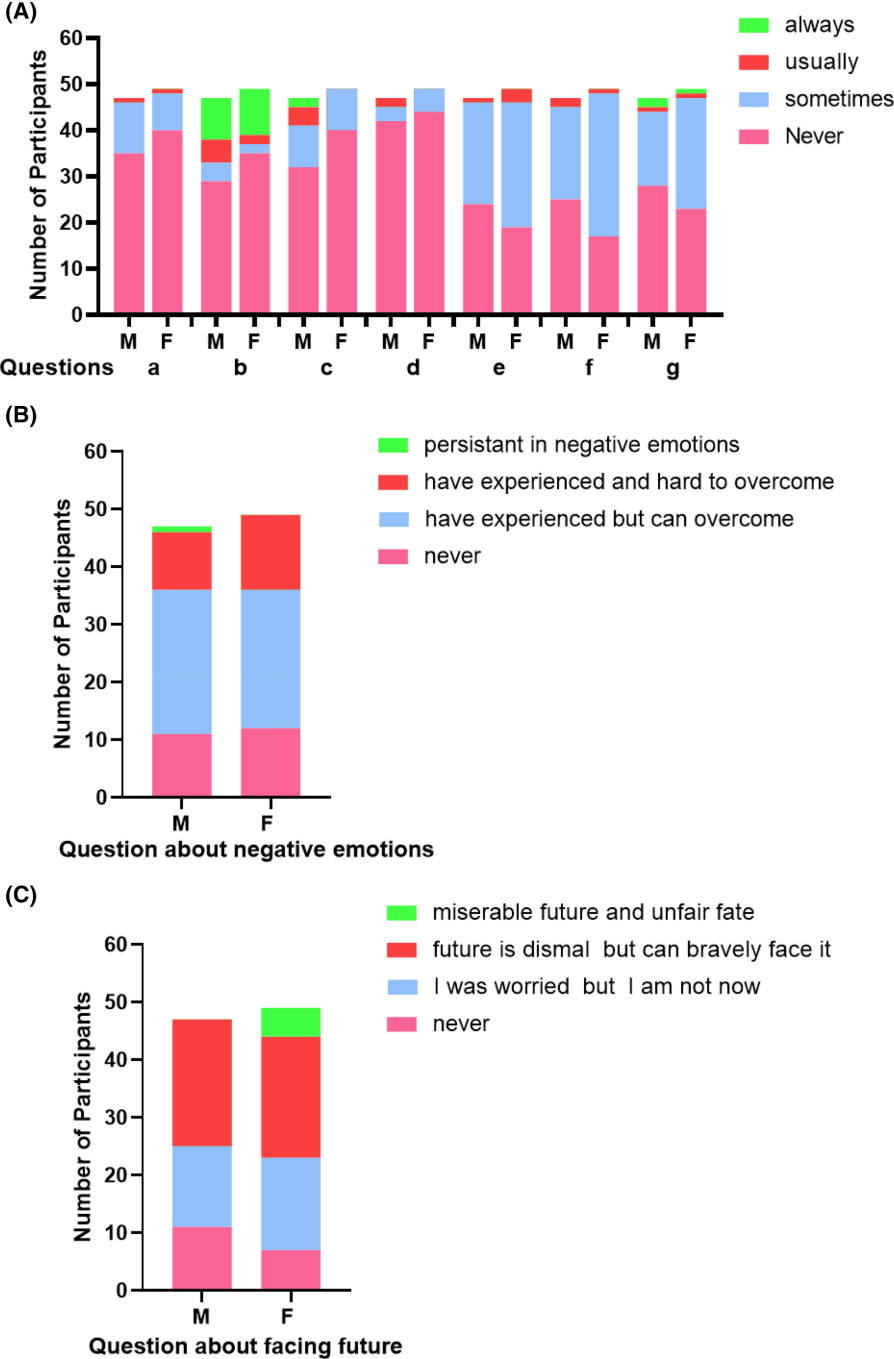
(A) 96 participants responses to the following seven questions. a. Do you feel frustrated at fighting your diseases? b. Do you think HSCT is not helping? c. Do you think therapeutic effect is much worse than you expected? d. Do you regret receiving HSCT? e. Do you live in fear of death? f. Do you worry about disease deterioration? g. Do you feel pain? (B) 96 participants responses to the following question: have you ever experienced negative emotions like confusion, distress, or even despair after allo‐HSCT? (C) 96 participants responses to the following question: do you worry about your future?

The participants completed inquiries about transplantation evaluation and emotion states. Although they all successfully received allo‐HSCT, quite a few survivors had doubts about curative effects (Figure 1A). 21.9% survivors sometimes or usually felt frustrated. Up to 19.8% survivors always thought HSCT did not help. 25.1% thought the effect of allo‐HSCT was worse than expected. However, 89.6% survivors never regretted receiving HSCT. More than half of the survivors sometimes or usually feared of death, worried about disease deterioration, and felt pain. More female survivors sometimes had fears of disease deterioration than male (63.3% vs. 42.6%, *p* = 0.042).

**FIGURE 2:**
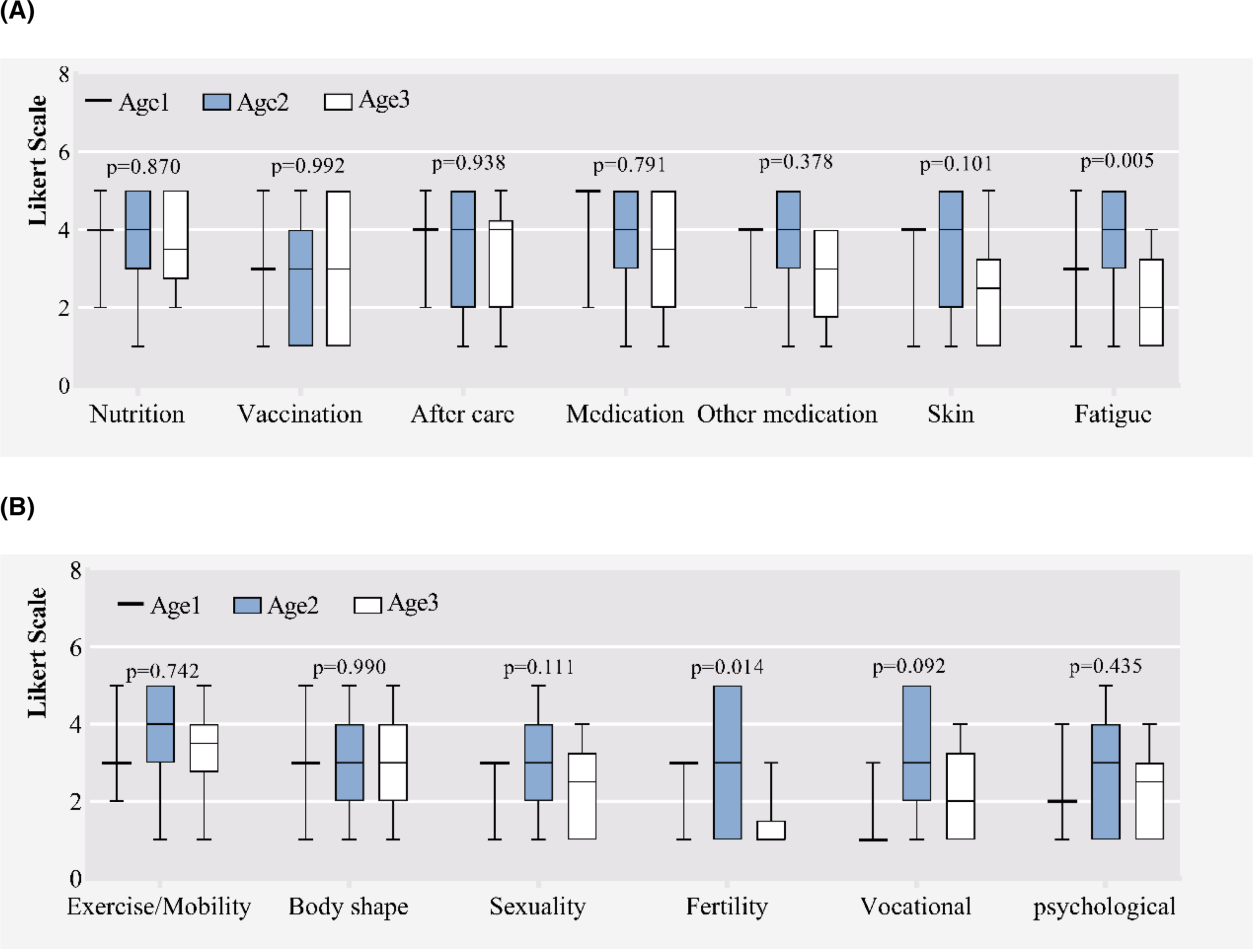
Different Informational needs according to age groups. Age1: <18 years. Age2: between 18 and 49 years. Age3: ≥50 years. (A) Medical issues. (B) Psychosocial issues. The box‐plot shows the maximum, minimum, median and interquartile of the data. Medication: medication of hematological disease; other medication: medication of non‐hematologic diseases.

75.0% survivors experienced less‐severe or severe negative emotions and went through a very difficult time (Figure 2B). Reasons for negative emotions included unsatisfied physical recovery; economic, work or life pressure; strained family relationships; fear of relapse and low self‐esteem. When asked “when your emotion reached the lowest point”, 31 survivors answered the question, and 23 reported the lowest point was within the first 6 months following allo‐HSCT, when complications like GVHD occurred. Negative emotions usually lasted from 2 months to many years.18 survivors reported they tried to improve their emotional states: only 4 survivors sought psychotherapy, 12 chose to self‐regulate, and 2 only used hypnotics.

When asked “do you worry about your future”, only 23.4% male survivors and 14.3% female survivors denied; Nearly half of the survivors thought their futures were dismal but they would face the future bravely.10.2% female survivors thought their future was miserable and could not help complaining about the unfair fate.

### 3.5 Medical and psychosocial needs

81.3% survivors reported unmet needs on financial assistance, psychological assistance and social care. When asked “what kind of supports do you want from doctors”, 44.8% survivors reported they needed knowledge related to medication, aftercare nutrition, the do's and don'ts in daily life, and 24.0% wanted compassionate physicians: willing to listen and communicate, showing understanding for the feelings of patients, and being caring and supportive. All patients were asked to rate their medical and psychosocial needs, ranging from very low to most intensive. In all issues, the topics of fatigue, nutrition, hematological disease treatments were most important. These were followed by physical activity and exercise, treatment of non‐hematologic diseases, aftercare, skin/alopecia, vaccination, body shape, sexuality, vocational assistance, fertility and psychological counseling.

**FIGURE 3:**
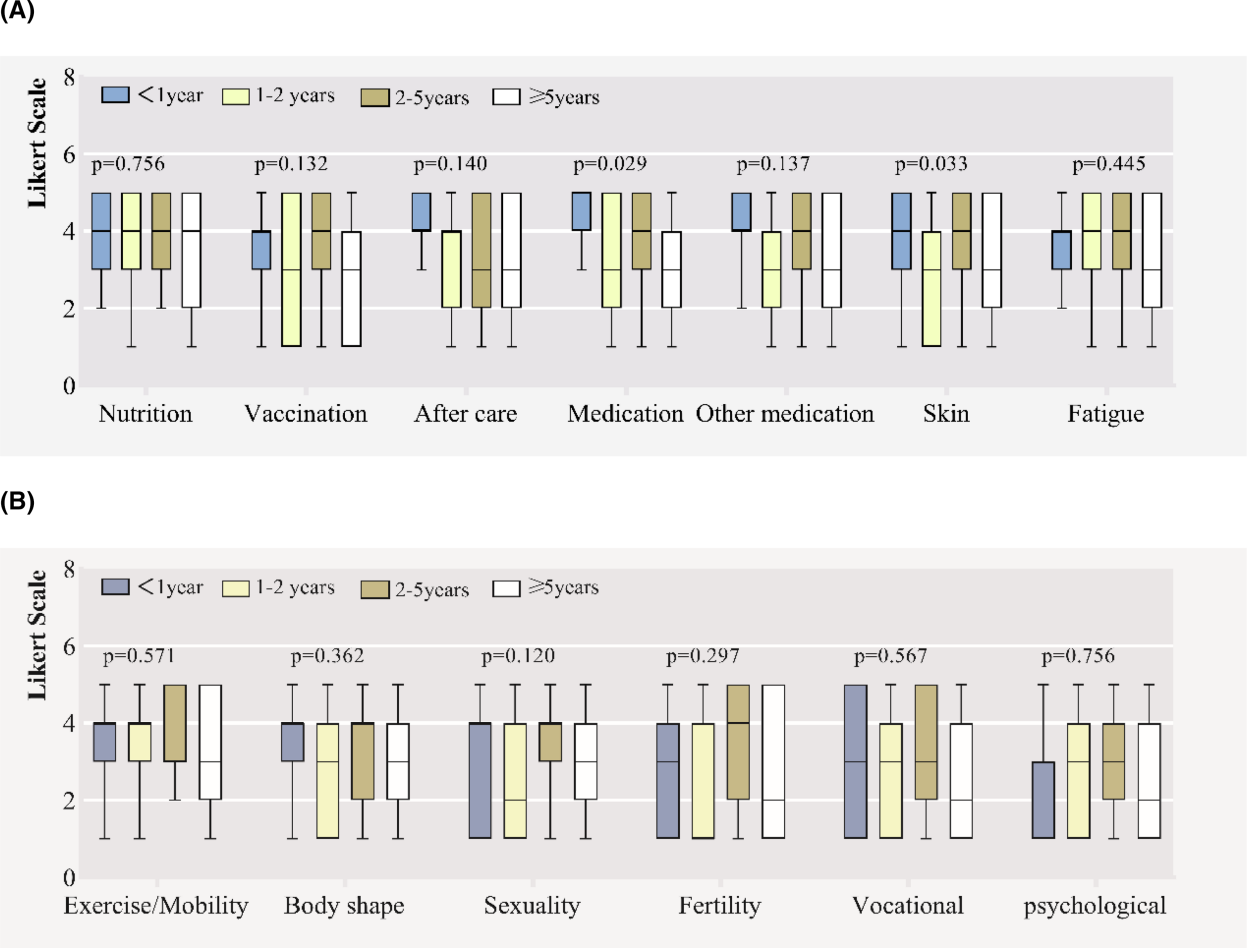
Different Informational needs according to time since hematopoietic stem cell transplantation. (A) Medical issues. (B) Psychosocial issues.

**FIGURE 4:**
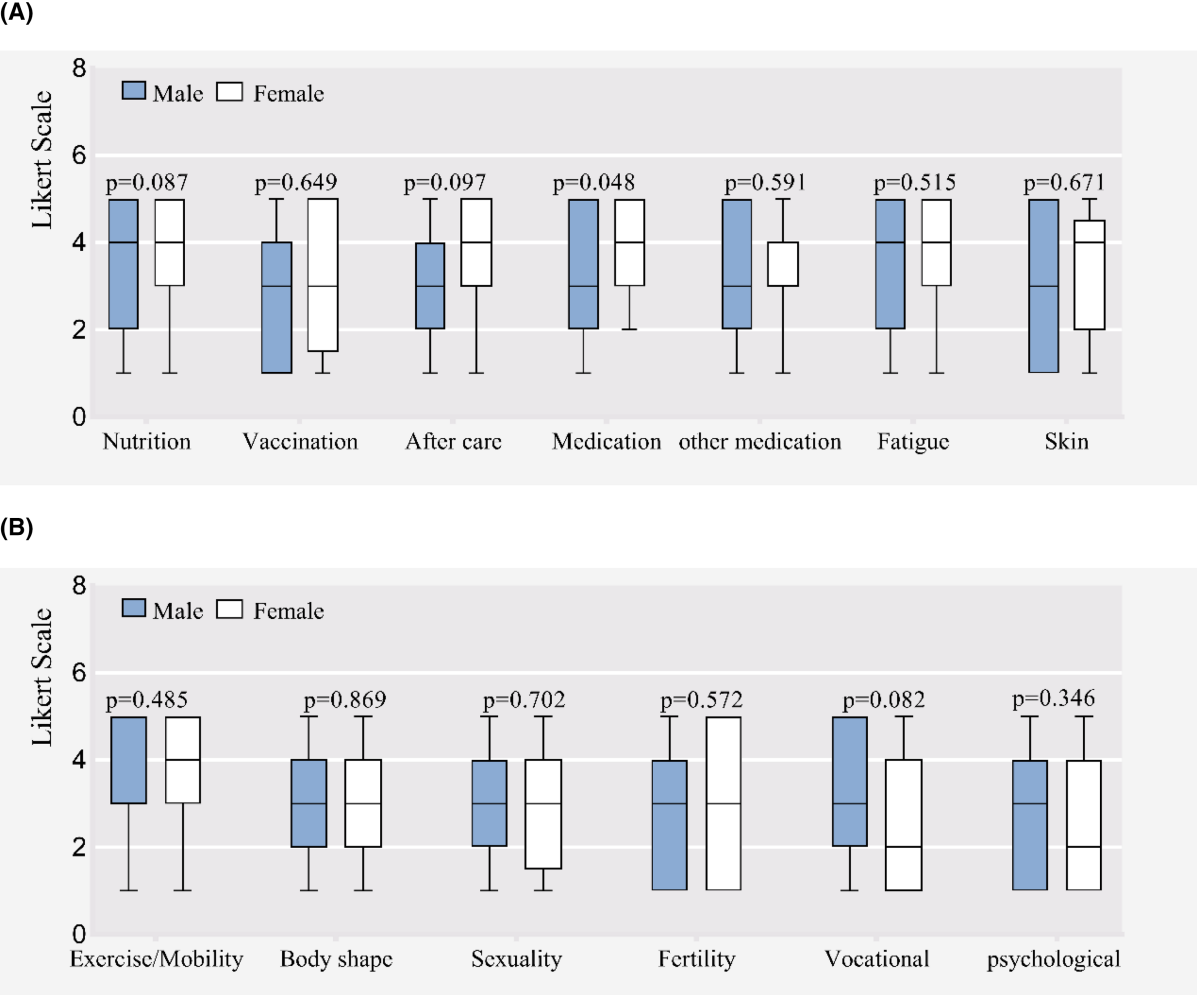
Different Informational needs according to gender groups. (A) Medical issues. (B) Psychosocial issues.

Figures 2–4 represent differences in informational needs according to age, time since HSCT and gender. Younger survivors (18–50 years) rated coping strategies for fatigue and fertility two points higher than elderly survivors (≥50 years) (median grade 4 (3–5) vs. grade 2 (1–3), *p* = 0.004; median grade 3 (1–5) versus grade 1 (1–1), *p* = 0.012). They rated nutrition, physical activity and exercise, skin, sexuality and vocational assistance higher, although without significant differences (Figure 2A,B). Patients surviving within 1 year since HSCT rated treatment information of hematological disease higher (*p* = 0.024). Patients surviving between 2 and 5 years rated informational needs regarding skin/alopecia higher (*p* = 0.033) (Figure 3A,B). Except medication information of hematological disease, there were no significant differences between the gender groups (see Figure 4A,B).

When asked about the preferred channel of information, the vast majority answers were WeChat public account and WeChat applet (36.5%), one‐to‐one conversation (34.4%), mobile interactive platform (15.6%), and checking interested knowledge on related websites by oneself (11.5%).

## 4 DISCUSSION

Published researches demonstrate that moderate to severe depressive symptoms^16^, ^17^ are common psychiatric comorbidities after HSCT. In our study, 55.2% allo‐HSCT recipients reported mild to severe depression based on BDI‐13, but only 4.2% had been clinically diagnosed with depression. This discrepancy suggested most patients were not aware that they needed to seek professional psychiatric help, or that their doctors did not communicate this to them. It could not exclude the possibility some of them might deliberately ignore their own psychological issues because of fear of stigmatization.^18^ However, our study revealed that up to 75.0% of survivors admitted to experiencing negative emotions, ranging from less severe to severe. The lowest point of these emotions commonly occurred within the first 6 months following allo‐HSCT, and they could persist for several months to many years. Quite a few survivors preferred self‐regulation over seeking help from a psychologist. Therefore, depression post‐allo‐HSCT might be substantially underestimated by patients themselves and our doctors, and should be paid an increasing attention to. Previous research demonstrated there is a strong evidence of a link between female sex and depression.^19^ Our study also showed higher proportions of female survivors had mild depression than male survivors, although there was no significant difference, which might be possible that the sample size was not large enough to be significant.

It has been demonstrated there is a strong and significant association between sleeping disorder, nutritional status and depressive disorder.^11^, ^18^, ^19^ Trouble sleeping, loss of appetite and malnutrition are frequent consequences of allo‐HSCT in survivors experiencing depression, and may occur several months or years after HSCT.^20^ In this study, 33.3% survivors had poor or awful sleep quality, 31.3% were underweight, and low body weight was more prominent in female. This phenomenon is rarely explored before. Our findings support the need for specialized nutritional care post‐allo‐HSCT, especially for female survivors.

In our study, 11.5% survivors' objective health states were unsatisfied based on ECOG performance status (ECOG ≥2), without significant difference between genders. However, more male survivors thought they were in very good health states, correspondingly, more female survivors assessed themselves in unsatisfied health states and need a caregiver sometimes. Therefore, despite not facing more physical problems, female survivors probably did feel more psychological pressure, which is consisted with Heinonen's research:^21^ female sex are associated with poorer quality of life. Gender differences exist among allo‐HSCT recipients need to be addressed when designing post‐HSCT intervention programs.

We further investigated what factors could affect the level of depressive disorder and trouble sleep. We found younger adults, single marital status (including divorced or widowed status), no or low dose of ATG, less than 5 years post‐HSCT, cGVHD, ECOG 2–4 were associated with depression severity based on BDI‐13; younger adults, chronic GVHD, ECOG 2–4 were associated with troubling sleep. ECOG 2–4 was strongly associated with both depression and sleeplessness, supporting the accepted opinion that poor health is likely to cause depressive symptoms. cGVHD is also an identified susceptible factor of poor quality of life.^21^, ^22^, ^23^ Following allo‐HSCT, up to 66% of patients could develop cGVHD.^24^, ^25^ cGVHD remains a major factor contributing to transplantation‐related morbidity and mortality, and poor quality of life. There is moderate to strong evidence that cGVHD predict post‐transplant psychological distress.^16^, ^19^, ^26^ In our study, cGVHD affected 33.3% HSCT survivors, and had an independent negative association with not only depressive disorder but also troubling sleep. Previous findings on the effect of age seems inconsistent: some study demonstrated younger age often predicted better physical well‐being in allo‐HSCT patients;^27^ another study showed younger age predicted impaired psychological functioning.^28^ An explanation for younger age being associated with depressive disorder and troubling sleep is that younger patients might experience more competing demands, face heavier responsibilities and hold higher expectations compared with older patients, potentially leading to a perception of poorer abilities.^28^, ^29^ Our study showed divorced/ widowed/ separated survivors were prone to depression, which is consistent with the common phenomenon:^30^ There is a strong association between marital status and psychological disorder, and separated/divorced marital status were consistently associated with depression. We found “less than 5 years since HSCT” was also the susceptible factor of depression, the reason might be allo‐HSCT recipients experienced higher symptom burden and worse physical functioning during and after the process of transplantation, so recipients' physical and psychologic condition declined rapidly, and gradually recovering toward pre‐HSCT levels in the years following transplantation.^4^, ^31^ Our results showed the dose of ATG was negatively associated with depression. ATG has consistently demonstrated efficacies in GVHD prophylaxis.^32^ No or low dose of ATG might increase the risk of chronic GVHD, then indirectly cause depression.

Allo‐HSCT survivors commonly have bias in the perception of transplant effects, which affect their psychological states. Overestimation of the benefits and underestimation of the morbidity and mortality related to HSCT are regular psychological reactions.^33^ Our data also showed more than 20% survivors did not satisfied with allo‐HSCT treatment effect. We then asked HSCT survivors some custom questions to examine their psychological status, and we found a special phenomenon. For questions about treatment evaluation, such as, “Do you feel frustrated at fighting your diseases?”, or” Do you think HSCT is not helping?” or “Do you think therapeutic effect is much worse than you expected”, female survivors tended to be more tolerant than male survivors: higher proportions of female survivors answered “never”. However, when asked how to face the uncertain outcome, such as “Do you live in fear of death?”, or “Do you worry about disease deterioration?”, or “do you worry about your future?”. female survivors seemed to be more pessimistic and more worried. Although without significant difference, gender seems to be an underlying influence on perceptions and concerns of allo‐HSCT.

Our study elicited four findings in informational need: (1) The majority of patients report unmet needs on physical and psychosocial aspects. (2) Nutrition information is the most important topic, followed by information on medication and fatigue. (3) Younger patients may require more information on fertility and fatigue issues, they were more interested in information regarding nutrition, sports, skin, sexuality and vocational assistance. (4) WeChat public account and WeChat applet, Mobile interactive platform and one‐to‐one conversation were the favorite channel for information.

Loss of appetite, nausea and vomiting would be the common issues during and after allo‐HSCT. Patients suffering from cGVHD may experience progressive emaciation. Therefore, nutrition is one of the most concerned issues. In our study, younger patients emphasized a higher level of informational need on the topic of nutrition, which is consistent with previous reports.^1^, ^34^ It is important to provide them more nutritional knowledge. Oncological chemotherapy treatment affects the fertility and sexual function of patients. The myeloablative conditioning can cause damage to the reproductive organs. Male patients may also have hormonal abnormalities and sexual dysfunction, such as retrograde ejaculation or infertility.^35^ How to help the younger patients to preserve their fertility and sexual function is an important issue. Fatigue is one of the most common symptoms experienced by patients post‐HSCT.^36^ In our study, younger patients expressed a higher demand for information on fatigue, which is in line with some previous study,^37^ but conflicts with the other.^1^ The desire for overcoming fatigue in younger survivors may due to higher employment pressure, social and family duty, self‐demanding.

Interestingly, we found a low demand of information on psychological counseling, which is consistent with the low proportion of patients aware that they needed to seek professional psychiatric help. However, the proportion of depression was high according to the score of BDI of our patients. Therefore, education for survivors in mental health is very necessary. We need to provide more counseling and knowledge on mental health for post‐HSCT survivors.

This retrospective study evaluates the psychological states and needs of patients post‐allo‐HSCT only at a single time point; and losts the data of nonparticipants, including some that may be the most severe patients suffering from psychological problems. Therefore, prospective studies are needed to more accurately reflects the psychological states and needs of patients undergone allo‐HSCT. Further interventional clinical studies are also necessary to better manage and treat the survivors who develop psychological symptoms post‐allo‐HSCT.

In this study, we concluded that depression and poor sleep quality were major concerns troubling allo‐HSCT survivors, and quite a proportion of patients are not aware that they needed to seek professional psychiatric help. Clinicians should establish more appropriate survivorship care plans focusing on survivors' psychologic states, demands and need.

## AUTHOR CONTRIBUTIONS

**Sun Yanling:** Conceptualization (equal); data curation (equal); formal analysis (equal); investigation (equal); methodology (equal); project administration (equal); resources (equal); software (equal); validation (equal); visualization (equal); writing – original draft (equal); writing – review and editing (equal). **Yan Pu:** Conceptualization (equal); data curation (equal); formal analysis (equal); investigation (equal); methodology (equal); resources (equal); validation (equal); visualization (equal); writing – original draft (equal); writing – review and editing (equal). **Xu Duorong:** Investigation (equal); resources (equal); writing – review and editing (equal). **Zhong Zhiyong:** Conceptualization (lead); methodology (lead); writing – review and editing (equal). **Xu Ting:** Conceptualization (equal); methodology (equal); writing – review and editing (equal). **Xin Wenjun:** Conceptualization (lead); methodology (lead); project administration (equal); supervision (lead); writing – review and editing (lead). **Zhang Xiangzhong:** Conceptualization (lead); funding acquisition (lead); investigation (equal); methodology (lead); project administration (lead); resources (equal); supervision (equal); validation (equal); visualization (equal); writing – review and editing (lead). **Zhang Jingwen:** Conceptualization (equal); data curation (equal); formal analysis (equal); investigation (equal); methodology (equal); project administration (equal); resources (equal); software (equal); supervision (equal); validation (equal); visualization (equal); writing – original draft (equal); writing – review and editing (equal).

## CONFLICT OF INTEREST STATEMENT

All authors declare no conflict of interest.

## Data Availability

All relevant data are within the manuscript and its additional files.
